# Caring for Bierzo: A plan for improving motivation for health workers from Mental Health

**DOI:** 10.1192/j.eurpsy.2023.689

**Published:** 2023-07-19

**Authors:** J. M. Pelayo-Terán, Y. Zapico-Merayo, S. Vega-García, M. E. García-Llamas, Z. Gutiérrez-Hervás, A. Castro-Bayón

**Affiliations:** ^1^Psiquiatría y Salud Mental. Unidad de Calidad y Seguridad del Paciente, Hospital El Bierzo. GASBI. SACYL. CIBERSAM.; ^2^Área de Medicina Preventiva y Salud Pública. Departamento de Ciencias Biomédicas., Universidad de León; ^3^ Psiquiatría y Salud Mental, Hospital El Bierzo; ^4^ Psiquiatría y Salud Mental; ^5^Unidad de Calidad y Seguridad del Paciente, Hospital El Bierzo. GASBI. SACYL, Ponferrada (León), Spain

## Abstract

**Introduction:**

Motivation is an essential determinant of performance, particularly for those working in difficult conditions, such as the conditions facing many health workers. The relationship between motivation and performance is influenced by the health workers’ organizational environment and social context. Many intrinsic and extrinsic factors may influence the impulse to head for and maintain and effort to achieve the objectives of the organization these may include acknowledgements, status and incentives, but also auto efficacy perception, personal growth, welfare and physical and mental health.

In the last years and particularly during COVID-19 pandemic health organizations have suffered a crisis of lack of motivation and high turnover rates in health professional, particularly among highly specialized professions.

**Objectives:**

to develop a quality program to promote mental health and motivation, detect risk of mental disorders and improve communication skills in the health workers of the Health Area of El Bierzo (GASBI).

**Methods:**

A committee with four members form the psychiatry and mental health service, two from the Quality and Security Unit and 1 from the Occupational Risk Prevention Service analyzed the GASBI with a SWOT-CAME matrix analysis. Actions to be implemented were ordered with a Hanlon method score according to a representative sample of the employees of GASBI.

**Results:**

The CAME matrix recommended an offensive strategy, given a number of strengths and the opportunities for an administration sensible to new paradigms. The program proposed included 6 main lines (evaluation, access to mental health consultation, prevention of suicide behavior, resilience group, communication and relation groups and a group of actions to improve motivation and prevent burnout called “10 actions to dream together”, displayed in figures 1 and 2. The Hanlon classified suicide behaviour as the highest priority (score: 16,25 points), mental disorders the second (score: 13.75), then fatigue (13 p), burnout and resilience (12p) and the less prioritary was motivation (7 points). The project was displayed in a canvas business model (figure 3)

**Image:**

**Figure:**
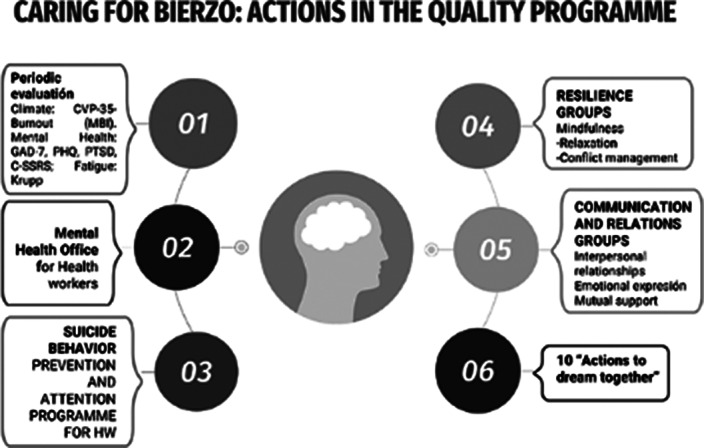

**Image 2:**

**Figure:**
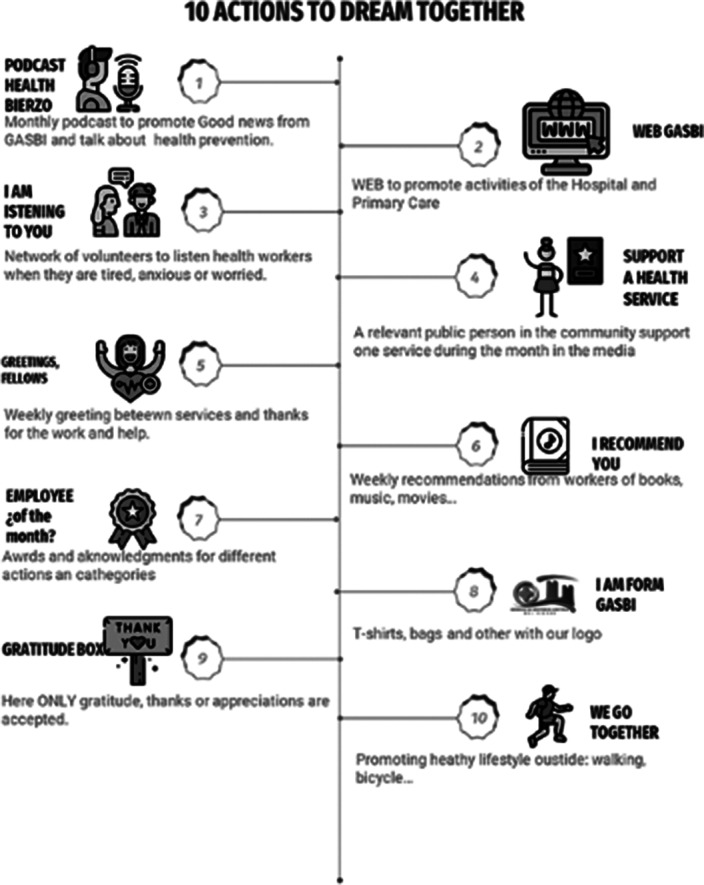

**Image 3:**

**Figure:**
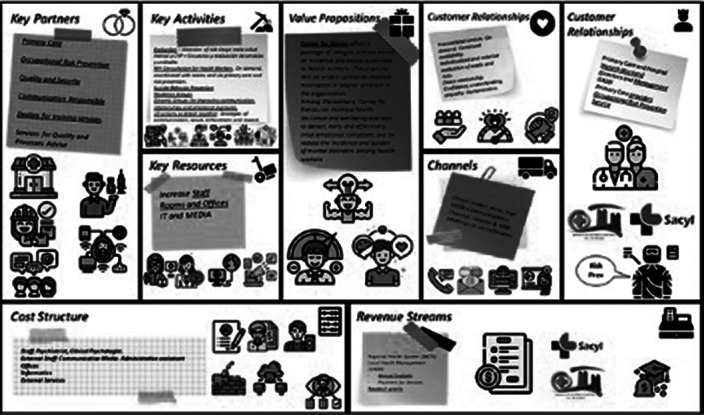

**Conclusions:**

Mental health, fatigue, burnout and motivation of health workers is a complex problem that affect health organizations and quality of services. Mental Health service have an important role in the promotion of wellbeing and prevent burnout in the health system.

**Disclosure of Interest:**

None Declared

